# Investigation of Spray Drying Parameters to Formulate Novel Spray-Dried Proliposome Powder Formulations Followed by Their Aerosolization Performance

**DOI:** 10.3390/pharmaceutics16121541

**Published:** 2024-12-01

**Authors:** Iftikhar Khan, Kaylome Edes, Ismail Alsaadi, Mohammed Q. Al-Khaial, Ruba Bnyan, Saeed A. Khan, Sajid K. Sadozai, Wasiq Khan, Sakib Yousaf

**Affiliations:** 1 School of Pharmacy and Biomolecular Sciences, Liverpool John Moores University, Liverpool L3 3AF, UK; 2 College of Pharmacy, University of Al Maarif, Al Anbar 31001, Iraq; mohammed.qahtan@uoa.edu.iq; 3 School of Life Sciences, Pharmacy and Chemistry, Kingston University, London KT1 2EE, UK; 4 Department of Pharmacy, Kohat University of Science and Technology, Kohat 26000, Pakistansajidsadozai@kust.edu.pk (S.K.S.); 5 Faculty of Engineering and Technology, Liverpool John Moores University, Liverpool L3 3AF, UK

**Keywords:** spray drying, mannitol, trehalose, lactose monohydrate, proliposome, dry powder inhaler

## Abstract

Background: Spray drying, whilst a popularly employed technique for powder formulations, has limited applications for large-scale proliposome manufacture. Objectives: Thus, the aim of this study was to investigate spray drying parameters, such as inlet temperature (80, 120, 160, and 200 °C), airflow rate (357, 473, and 601 L/h) and pump feed rate (5, 15, and 25%), for individual carbohydrate carriers (trehalose, lactose monohydrate (LMH), and mannitol) for 24 spray-dried (SD) formulations (F1–F24). Methods: Following optimization, the SD parameters were trialed on proliposome formulations based on the same carriers and named as spray-dried proliposome (SDP) formulations. Drug delivery of the formulations was assessed using a dry powder inhaler (DPI) in combination with a next-generation impactor (NGI). Results: Upon analysis, formulations F6 (SD-mannitol), F15 (SD-trehalose), and F20 (SD-LMH) demonstrated high production yields (84.01 ± 3.25, 72.55 ± 5.42, and 70.03 ± 3.39%, respectively), small particle sizes (2.96 ± 1.42, 4.55 ± 0.46, and 5.16 ± 1.32 µm, respectively) and low moisture contents (0.25 ± 0.03, 3.76 ± 0.75, and 1.99 ± 0.77%). These SD optimized parameters were then employed for SDP formulations employing dimyristoly phosphatidylcholine (DMPC) as a phospholipid and beclomethasone dipropionate (BDP) as the model drug. Upon spray drying, SDP-mannitol provided the highest production yield (82.45%) and smallest particle size (2.64 µm), as well as high entrapment efficiency (98%) and a high fine particle dose, fine particle fraction, and respirable fraction (285.81 µg, 56.84%, 86.44%, respectively). Conclusions: The study results are a promising step in the optimization of the large-scale manufacture of proliposome formulations and highlight the versatility of the instrument and variability of formulation properties with respect to the carriers employed for targeting the pulmonary system using dry powder inhalers.

## 1. Introduction

The pulmonary system is a non-invasive and historically used route for inhalation therapy. It offers a highly vascularized, large surface area (circa 100 m^2^), facilitating rapid absorption [1,2]. Recently popularized for both localized and systemic delivery, this route has allowed for the delivery of water-soluble active compounds with associated poor bioavailability when administered via alternative routes [3]. In the localized treatment of pulmonary diseases, formulations can be deposited directly onto the lung epithelium; this facilitates rapid onset of action, potentially negating drug degradation and resulting in lower dosing requirements. Beclomethasone dipropionate (BDP) is a synthetic steroidal drug delivered via inhalation for the prophylaxis of asthma symptoms. As a model drug, it has been employed in a myriad of micro- and nanoformulations. Recently, these formulations have gained popularity in encapsulating therapeutically active compounds for their delivery to the pulmonary system. These offer drug loading ability, biocompatibility, and biodegradability, preventing drug degradation and ensuring sustained release and formulation stability. Various lipid-based formulations offer these characteristics, delivering drugs to the pulmonary system, including transfersomes and protransfersomes [4], niosomes and proniosomes [5,6], liposomes and proliposomes [7,8], solid lipid nanoparticles [9], hybrid nanoparticles [10,11], microspheres [12], and nanostructured lipid carriers [13].

Liposomes have been extensively employed as a carrier system for delivering hydrophilic drugs (entrapped in the central core) and lipophilic drugs (entrapped in the vesicle’s concentric bilayers) [14]. However, it is established that liposomes are associated with instability during storage, commonly resulting in the leakage of entrapped drugs, vesicle aggregation, and fusion [15,16]. This instability is attributed to the oxidation and hydrolysis of liposomal phospholipids, which may curtail the shelf-life of liposome formulations. To curb the observed stability issues and capitalize on the potential of liposomes, proliposome formulations have been developed [17]. Proliposomes are powdered formulations comprising carbohydrate carriers (e.g., sucrose, sorbitol, lactose, and mannitol) loaded or coated with phospholipid, and can generate liposomes upon hydration. Production of proliposomes can be achieved through a plethora of methods, including the particulate-based slurry method, fluidized-bed coating [18] and freeze drying. However, these methods suffer from numerous drawbacks, including extended production times, as well as being associated with potential phospholipid and drug loss during the proliposome formulation process. The manufacture of spray-dried proliposome (SDP) [2,19,20] formulations offer select advantages, such as minimized wastage of drug and excipients. SDP formulations containing micronized particles can be delivered effectively via dry powder inhalers (DPIs).

Since 1970, DPIs have been commercially available for drug delivery targeting the pulmonary system [21]. DPIs are essentially breath-actuated devices and do not require a combination of coordinated press-and-breathe efforts. During inhalation or when using an artificial model, inspiratory air generates high turbulence from DPIs, resulting in the dry powder formulation being carried into the lungs. However, deposition of powder formulations using DPIs differs from device to device, as well as from formulation to formulation. There are also additional factors which can affect the dose of drug, including inspiratory flow rate, formulation flow properties, particle size, shape, and density, and inter-particulate interaction.

**Scheme 1:**
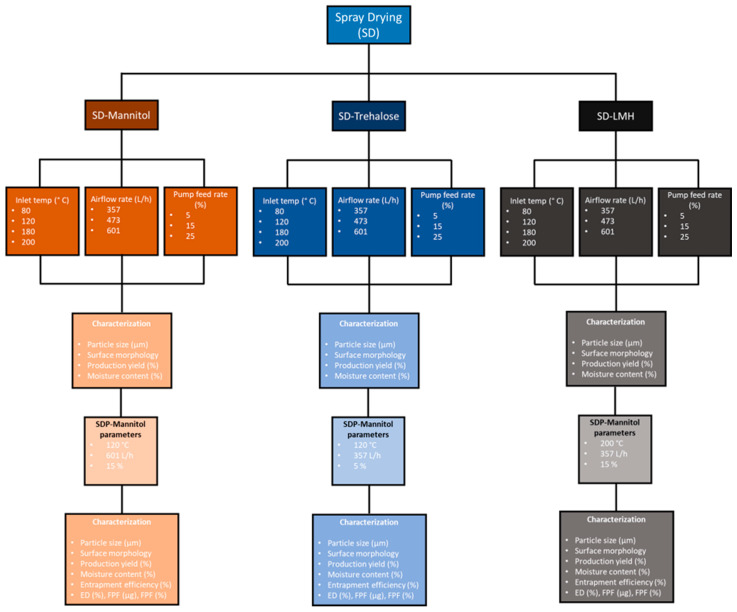
Representing the whole study from the parameter selection to spray-dried (SD) and spray-dried proliposome (SDP) formulation characterization.

In this study, novel spray-dried (SD) formulations were developed, optimized, and characterized based on the ideal parameters employed via spray drying in order to identify and specify parameters for small to large scale manufacturing as well as their ideal deposition in the pulmonary system for better patient outcome. These SDP formulations were prepared using beclomethasone dipropionate (BDP) as a model drug. Mannitol, trehalose, and LMH carriers were investigated for their potential use as carbohydrate carriers in a unique combination of spray drying parameters, including inlet temperatures, airflow rates, and pump feed rates; this was followed by characterization, namely of production yield, particle size, moisture content, particle morphology, particle nature (crystalline/amorphous), and entrapment efficiency. Additionally, following optimization, the novel SDP formulations were studied for their aerosolization performance using a DPI in combination with an artificial lung model (i.e., a next-generation impactor; NGI) to assess drug deposition. The full illustration of the study is provided in Scheme 1.

## 2. Materials and Methods

### 2.1. Materials

Mannitol, trehalose dihydrate, Tween 20, and beclomethasone dipropionate (BDP) were purchased from Sigma Aldrich, Gillingham, UK. Lactose monohydrate (LMH) was acquired from VWR Chemicals, Lutterworth, UK. HPLC-grade methanol and ethanol were purchased from Fisher scientific, Loughborough, UK. Dimyristoylphosphatidylcholine (DMPC) was procured from Lipoid, Steinhausen, Switzerland. Hydroxypropyl methylcellulose (HPMC) capsules (size 3) were gifted by Qualicaps, Madrid, Spain. Dry powder low-resistance inhalers (RS01; 4 kPa at 100 L/min) were a generous gift from Plastiape, Osnago, Italy.

### 2.2. Spray Drying of Mannitol, Trehalose, and LMH Carriers

Spray-dried (SD) powders of coarse carbohydrate carriers (i.e., mannitol, trehalose, or LMH) were prepared via a spray dryer (Büchi B-290 Mini Spray Dryer, Switzerland) with a high-performance cyclone, where four different inlet temperatures (80, 120, 160, and 200 °C), three different airflow rates (357, 473, and 601 L/h), and three different pump feed rates (5, 15, and 25%; 1.5, 4.5, and 7.5 mL/min) were used. These spray drying conditions were employed to prepare 24 SD formulations (Table 1). During preparation, each coarse carbohydrate carrier (mannitol, trehalose, or LMH) (2000 mg) was dissolved in 50 mL of solvent (first in 25 mL of water, followed by the addition of 25 mL of ethanol) to make a 4% w/v solution, which was SD using a 0.7 mm diameter spray dryer nozzle, systematically employing one factor from each of the inlet temperatures, airflow rates, and pump feed rates. The spray drying procedure was repeated for all coarse carbohydrate carriers, using each inlet temperature, airflow rate, and pump feed rate to prepare SD powders in order to determine the optimum parameters for formulation (Table 1) using spray-drying instruments. The following characteristics were analysed: particle morphology, production yield, particle size, moisture content, and crystallinity.

Post-spray drying, the SD powders were collected in a collection vial/chamber and the production yield determined with the help of the following Equation (1):

(1) $$Production\;Yield\left(\%\right)=\left(\frac{W_{f}{-W}_{e}}{W_{i}}\right)\times 100$$

where W_f_ is the final mass of the powder collected after spray drying, W_e_ is the mass of the empty collecting chamber, and W_i_ is the initial mass of coarse carbohydrate dissolved in a solvent before spray drying.

### 2.3. Spray Drying Proliposome (SDP) Formulations

The following selection of SDP formulations was based upon the spray drying results from the method outlined in Section 3.2 (Table 1). For SDP formulations, DMPC (281.69 mg) and BDP (28.17 mg) were dissolved in ethanol (25 mL). Coarse mannitol (1690.41 mg) was dissolved in distilled water (25 mL). Both solutions were mixed via vortex mixing (Fisons WhirliMixer, Fisons Scientific Equipment, UK) for 2 min at 100 RPM to achieve optimum mixing in order to obtain a 4% *w*/*v* formulation, followed by spray drying.

For SDP-mannitol formulations, an inlet temperature of 120 °C, an airflow rate of 601 L/h, and a pump feed rate of 15% were used, whereas for the SDP-trehalose formulation, an inlet temperature of 120 °C, an airflow rate of 357 L/h, and a 5% pump feed rate were employed. In the SDP-LMH formulation, a 200 °C inlet temperature, 357 L/h airflow rate, and 15% pump feed rate were used. The resultant SDP formulations were collected in airtight containers and stored in a desiccator for further investigation. The same procedure was repeated in triplicate for all SDP formulations.

### 2.4. Surface Morphology of Coarse Carriers, SD Formulations, and SDP Formulations via Scanning Electron Microscopy (SEM)

Particle surface morphology and particle size (around 100 particles were measured) of coarse carbohydrate carriers (mannitol, trehalose, or LMH), spray-dried (SD) formulations (when prepared using various parameters of the spray-drying instrument, e.g., inlet temperatures, airflow rates, and pump feed rates), and spray-dried proliposome (SDP) formulations were examined and compared using scanning electron microscopy (SEM) (EFI Quanta 200, Brno, Czech Republic). Each sample was placed onto carbon-coated aluminium stubs and coated with a film of gold using a sputter coater (Emitech K550x Gold Sputter Coater, Kent, UK). Samples were examined and a number of images were taken.

### 2.5. Powder X-Ray Diffraction (PXRD) Studies

Coarse carbohydrate carriers (mannitol, trehalose, or LMH) and SD and SDP formulations were studied using powder X-ray diffraction (PXRD) (Rigaku Miniflex, Rigaku Ltd., Tokyo, Japan) with Cu-Kα (λ = 0.154 nm) as an X-ray source. Samples were loaded onto silicon standard sample holders and scanned with 30 kV of voltage and 15 mA of current. All samples were scanned at 2θ between the angular ranges of 5–55° using a scan rate of 2°/min. The peaks from coarse carbohydrate carriers and SD and SDP formulations were examined and compared with respect to their crystalline and amorphous forms.

### 2.6. Moisture Analysis via Thermogravimetric Analysis (TGA)

Thermogravimetric analysis (TGA Q50, TA Instruments, New Castle, DE, USA) was performed on coarse carbohydrate carriers and SD and SDP formulations to determine their moisture content. Samples weighing between 5–10 mg were placed onto a platinum pan and then loaded into the furnace. The TGA was set to a ramp increase from 25 °C to 250 °C, at a set rate of 20 °C/min, in order to observe any changes in the mass of the loaded samples.

### 2.7. In Vitro Performance of SDP Formulations Using a Next-Generation Impactor (NGI)

Prior to operation, the NGI components (Copley Scientific Limited, Nottingham, UK) and plates were washed (initially with water and then with methanol) and dried. NGI plates were coated with 1% (*v*/*v*) of Tween 20 with methanol and left to dry (3 h), forming a layer over the plate to curb the bouncing of particles during aerosolization. The NGI was coupled with a Copley TPK 2000 critical flow controller and a Copley HCP5 vacuum pump (Copley Scientific, Nottingham, UK). The air flow rate was measured and adjusted to 60 L/min prior to each experiment, employing a Copley DFM 2000 flow meter (Copley Scientific, Nottingham, UK). For the NGI at a 60 L/min flow rate, the effective aerodynamic cut-off diameter for each impaction stage was calibrated by the manufacturer and stated as Stages 1 (8.06 μm), 2 (4.46 μm), 3 (2.82 μm), 4 (1.66 μm), 5 (0.94 μm), 6 (0.55 μm), 7 (0.34 μm), and micro-orifice collector (MOC) (<0.34 μm).

Five hydroxypropyl methylcellulose (HPMC) hard capsules (size 3) were used. Each capsule was filled with 20 mg of the SDP-mannitol formulation. The filled capsules were then individually loaded into the mono-dose inhaler (DPI), which was then tightly fitted with the aid of a mouthpiece adapter in the throat section (i.e., induction port) of the NGI. The NGI was operated in conjunction with a controlled flow rate (60 L/min) and a delay time of 15 s (NGI flow controller) before the capsules were pierced within the DPI (using the device’s two pins). The particles of the SDP-mannitol formulation were then drawn into the NGI plates for 4 s. This was repeated for all the filled capsules using the SDP-mannitol formulation. Following the delivery of 100 mg of the SDP-mannitol formulation, the concentration of BDP in each stage was determined via HPLC by washing the relevant stage with a mixture of methanol and water (75:25 *v*/*v*). The same procedure was repeated for SDP-trehalose and SDP-LMH formulations in triplicate. The emitted dose (ED; Equation (2)), fine particle dose (FPD; Equation (3)), fine particle fraction (FPF; Equation (4)), and respirable fraction (RF; Equation (5)) were also calculated, as follows:

(2) $$ED\left(\%\right)=\left(\frac{Initial\;mass\;in\;capsules-Final\;mass\;remaining\;in\;capsules}{Initial\;mass\;in\;capsules}\right)\times 100$$

(3) $$FPD\left(mg\right)=Mass\;of\;particles\;on\;Stage\;2\to Stage\;7$$

(4) $$FPF\left(\%\right)=\left(\frac{FPD}{Initial\;mass\;loaded\;into\;capsules}\right)\times 100$$

(5) $$RF\left(\%\right)=\left(\frac{Mass\;of\;particles\;on\;Stage\;2\to 7}{Total\;particle\;mass\;on\;all\;stages}\right)\times 100$$

The mass median aerodynamic diameter (MMAD) and the geometric standard deviation (GSD) were calculated using Copley Inhaler Testing Data Analysis Software (CITDAS) Version 3.10.

### 2.8. Separation and Entrapment Efficiency of BDP

Liposomes were generated in water from SDP powder formulations (150 mg/5 mL), followed by 1 h of annealing to form stable vesicles. For the total drug, 1 mL from the liposome suspension was separately dissolved and diluted in 10 mL of methanol, and the presence of BDP was quantified by employing HPLC (Agilent 1200 HPLC instrument, Waldbronn, Germany). For the entrapped BDP, 0.5 mL of the liposome suspension was transferred to a Millipore filter (5 KDa) (Fisher Scientific, UK), and bench centrifugation (Spectrafuge 24D, Labnet International, Edison, NJ, USA) was conducted for 30 min using a centrifuge speed of 15,100× *g*. This process helped to retain and separate BDP-entrapped liposomes (i.e., vesicles were retained by the filter due to their size) and only the unentrapped drug (i.e., free drug) passed through the filter. The filtered unentrapped BDP was then diluted with 1 mL of methanol to determine the entrapped BDP in liposomes using the following Equation (6):

(6) $$Entrapment\;efficiency\left(\%\right)=\left(\frac{Total\;drug-Unentrapped\;drug}{Total\;drug}\right)\times 100$$

Quantification of BDP was achieved via HPLC, using methanol and water (75:25% *v*/*v*) as a mobile phase with a flow rate of 1.7 mL/min, and a detection wavelength of 239 nm. HPLC temperature was set at 40 °C, with an injection volume of 20 µL. The HPLC column used was 5 µm, 15 cm × 4.6 mm C-18 (Agilent Technologies, Santa Clara, CA, USA). For the quantification of unknown BDP concentrations, a calibration curve of BDP (0–80 µg/mL) was constructed.

### 2.9. Statistical Analysis

Statistical analysis was conducted on the data obtained using one-way analysis of variance (ANOVA) or Student’s *t*-test for comparing more than two groups or two sets of data, respectively. All experiments were performed in triplicate. *p* values less than 0.05 between groups was deemed statistically significant.

## 3. Results and Discussion

### 3.1. Effect of Inlet Temperature on the Physicochemical Properties of Spray-Dried (SD) Formulations

Four different inlet temperatures (80, 120, 160, and 200 °C) were employed to identify their effect and to determine the ideal/optimum inlet temperature for the following spray-dried (SD) formulations: SD-mannitol F1–F4; SD-trehalose F9–F12; and SD-LMH F17–F20 (see Table 1). These inlet temperatures were employed for all three coarse carbohydrate carriers (mannitol, trehalose, and LMH) using a constant airflow rate and pump feed rate of 357 L/h and 15%, respectively (Section 2.2).

#### 3.1.1. Effect of Inlet Temperatures on Particle Morphology and Particle Size

**Figure 1:**
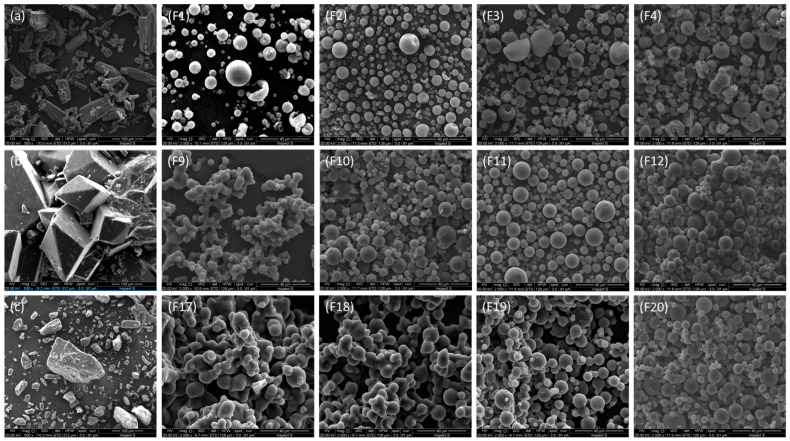
SEM images of three coarse carbohydrate carriers, namely (**a**) mannitol, (**b**) trehalose, and (**c**) LMH, followed by the effect of four inlet temperatures (80, 120, 160, and 200 °C) employing a spray-drying instrument on spray-dried mannitol formulation (F1, F2, F3, and F4), spray-dried trehalose formulations (F9, F10, F11, and F12), and spray-dried lactose formulations (F17, F18, F19, and F20), respectively. These images are typical of three such different experiments.

**Figure 2:**
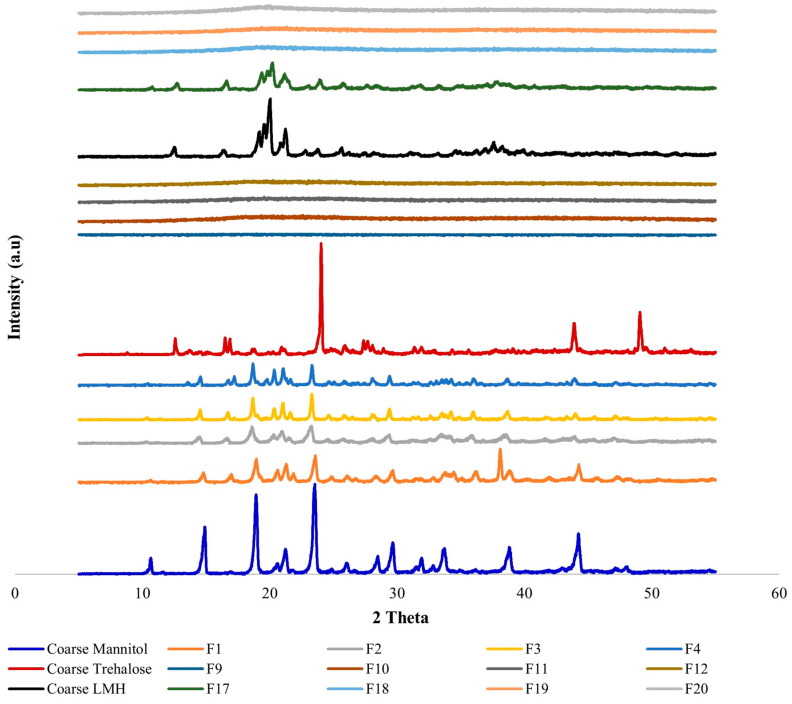
PXRD spectra of coarse carbohydrate carriers, including mannitol, trehalose, and LMH along with their corresponding spray-dried powder formulations of SD-mannitol (F1, F2, F3, and F4), SD-trehalose (F9, F10, F11, and F12), and SD-LMH (F17, F18, F19, and F20). These data are typical of three such different experiments.

Upon analysis of SEM images, it was observed that the coarse mannitol particles were oblong in shape (Figure 1a), whereas post-spray drying, spherical particles were observed. Using lower inlet temperatures of 80 and 120 °C, SD particles were noted to be spherical in shape (Figure 1; F1 and F2) and were deemed to be deposited in the peripheral airways. An ideal SD powder should be monodispersed with highly spherical particles, with an aerodynamic diameter range of 0.5–5 μm. The particle sizes of SD-mannitol formulations were found to be smaller for F2 formulations (i.e., 4.58 ± 0.91 µm; at an inlet temperature of 120 °C) when compared to counterpart SD-mannitol formulations (i.e., F1, F3 and F4) (Table 2). However, as the inlet temperature was increased to 160 and 200 °C, the glass transition temperature of mannitol (i.e., 167–170 °C) was reached/exceeded, resulting in fragmented particles (Figure 1; F3 and F4), which may be related to the crystalline profile of SD-mannitol formulations [1] (Figure 2; F1–F4). Additionally, breaking strength is an important indicator of particle integrity. Upon spray drying of these formulations at a constant solute concentration using higher inlet temperatures (160 and 200 °C), the droplets formed are converted into particles, which are hollow with a thin shell. These particles are more prone to collapse, and may break, presumably leading to the formation of debris of a smaller size [22]. Moreover, the higher inlet temperature increased the drying rate, which led to the formation of larger and hollow particles in general. This may be attributed to the establishment of the particle structure early with increasing air-drying temperature, hence preventing their shrinkage and resulting in larger particles [23].

Coarse trehalose particles were noted to be large and irregular or diamond in shape (Figure 1b), whereas post-spray drying, SD-trehalose particles were fused together (due to their amorphous nature) as agglomerates at 80 °C (Figure 1; F9). It is also suggested that, at lower inlet temperatures, particles may contain high moisture content (fully reaching the first stage (droplet evaporation) and second stage (wet particle formation and wet particle drying) of spray drying and partially reaching the third stage (final drying of residual moisture)) (Table 2), and, thus, these particles possess a tendency to agglomerate/fuse together. However, further increases in inlet temperature (i.e., 120, 160, and 200 °C) had no observable effect on the surface morphology of trehalose (Figure 1; F10, F11 and F12), and separate particles were observed. Amongst formulations F9–F12, only F10 was noted to possess a smaller particle size (4.74 ± 0.95 µm) than the respective SD-trehalose formulations (Table 2).

SEM images of the coarse LMH exhibited irregular particles with a high variance in size (Figure 1c). This may be related to the inlet temperature of the SD-LMH formulations, which was within the range of the glass transition temperature of the carbohydrate carrier (LMH) (i.e., 101–110 °C); formulations F17 and F18 prepared at the inlet temperatures of 80 and 120 °C, respectively, exhibited agglomeration (Figure 1). Moreover, when SD particles in the presence of high moisture content are warmed closer to their glass transition temperature, the particle surface becomes viscous, forming bridges with adjacent particles, causing agglomeration. This was observed in SD formulations F9, F17, and F18. However, as the inlet temperature of SD-LMH was further increased (i.e., 160 and 200 °C), round and spherical particles were obtained (Figure 1; F19 and F20), where formulation F20 demonstrated a smaller particle size (i.e., 5.16 ± 1.23 µm) than the F19 formulation (Table 2). Overall, based on surface morphology and particle size, SD formulations F2 (mannitol-based), F10 (trehalose-based), and F20 (LMH-based) were found to possess smooth surfaces, spherical shapes, and smaller particles.

#### 3.1.2. Effect of Inlet Temperatures on Production Yield, Moisture Content, and Particle Crystallinity

Upon analysis of the production yield using various SD-mannitol formulations (i.e., F1–F4), F1 exhibited a significantly lower production yield (*p* < 0.05) than formulations F2, F3, and F4 (Table 2). SD-mannitol formulations demonstrated an increasing trend of yield from F1 through to F2 and F3 (38.17 ± 2.61, 60.73 ± 4.12, and 63.64 ± 2.35%, respectively); this corresponded to increases in inlet temperature from 80 to 120 to 160 °C. However, at 200 °C, formulation F4 exhibited a lower production yield of 55.57 ± 6.64% (Table 2). This lower production yield may be attributed to the degradation of the mannitol carrier, as the melting point of the carrier (167–170 °C) has been exceeded. This may lead to particle fragmentation (Figure 1; F4), which is related to the crystalline nature of mannitol particles (Figure 2), and, hence, particle remnants escape from the collection vessel (and deposit on the filter, preventing their collection) during the spray drying process, resulting in a lower production yield. Furthermore, formulations F1–F4 exhibited intense peaks, demonstrating their crystalline nature (Figure 2). It is noteworthy that crystalline materials have greater stability than amorphous materials [24]. However, if they possess higher density, then they may possess a greater tendency to be deposited in the upper respiratory tract of the lungs.

Amongst the SD-trehalose formulations (F9–F12), F9 demonstrated a significantly lower (*p* < 0.05) production yield, and F12 showed a significantly higher (*p* < 0.05) production yield (Table 2). An increasing trend of production yield was observed across formulations F9 to F12 (14.63 ± 3.32 to 74.37 ± 4.33%), with increasing inlet temperature (from 80 to 200 °C) and decreasing moisture content (from 7.22 ± 1.13 to 1.75 ± 0.85%) (Table 2). Comparatively, a reduced yield was observed for formulation F9 at 80 °C, potentially attributed to inadequate drying temperatures, resulting in particles with a higher moisture content (7.22 ± 1.13%) compared to particles at higher inlet temperatures (Table 2). These results observed were additionally confirmed by research conducted by Sussich et al. [25], where the dehydration transition of dihydrate trehalose was noted to be between 91–97 °C. Moreover, coarse trehalose demonstrated intense peaks, indicating a crystalline structure, whereas SD-trehalose formulations (F9, F10, F11, and F12) showed amorphous characteristics at the four different inlet temperatures (Figure 2). Amorphous particles are desirable, as they generally provide a larger surface area and deeper lung deposition due to their high molecular mobility and low particle density. It is important to know that atomization of the carrier solution in the hot airflow during spray drying may lead to the formation of droplets. The evaporation of water induces a carrier/sugar concentration within droplets, and the carrier/sugar in the formulation solidifies via amorphous precipitation. Moreover, the carriers/sugars that contain a higher molecular weight with higher glass transition temperatures and low water contents become dry amorphous powders upon quick evaporation [26].

Similar to SD-trehalose formulations, SD-LMH formulations (F17–F20) displayed a trend where F17 demonstrated a significantly lower (*p* < 0.05) production yield and F20 exhibited a significantly higher (*p* < 0.05) production yield. This was again connected with increasing inlet temperature and decreasing moisture content (Table 2). Amongst the PXRD of SD-LMH formulations, formulation F17 was observed to be a crystalline powder, where the peaks were lower in intensity when compared to coarse LMH (Figure 2). This may be related to the lower inlet temperature (i.e., 80 °C), which was insufficient to evaporate water content from the SD formulations (Table 2), ultimately causing crystallization of the resultant powder [27]. Overall, the moisture content plays a vital role in determining shelf-life; as the moisture content decreases, the shelf-life of SD powders increases [28]. Based on the above characterization, the most ideal SD formulations manufactured via spray drying were F2 (SD-mannitol), F10 (SD-trehalose), and F20 (SD-LMH) using inlet temperatures of 120, 120, and 200 °C, respectively.

### 3.2. Effect of Airflow Rates on the Physicochemical Properties of Spray-Dried Formulations

Based on the aforementioned characterizations, SD formulations F2 (SD-mannitol), F10 (SD-trehalose), and F20 (SD-LMH) using inlet temperatures of 120, 120, and 200 °C, respectively, were selected. Therefore, their selected ideal inlet temperatures were kept constant. Additionally, pump feed rate was also kept constant at 15%, but for the determination of an ideal airflow rate, three different airflow rates (357, 473, and 601 L/h) were used for the SD formulation of mannitol (F2, F5, and F6), trehalose (F10, F13, and F14), and LMH (F20, F21, and F22) (Table 1).

#### 3.2.1. Effect Airflow Rates on Particle Morphology and Particle Size

**Figure 3:**
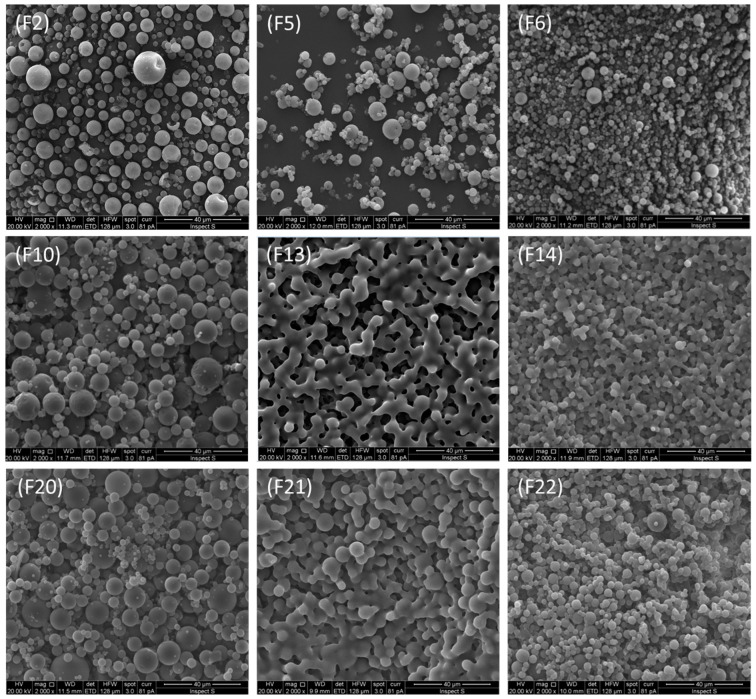
SEM images illustrating the effects of three airflow rates (357, 473, and 601 L/h) employing a spray-drying instrument on spray-dried mannitol formulations (F2, F5, and F6), spray-dried trehalose formulations (F10, F13, and F14), and spray-dried lactose formulations (F20, F21, and F22), respectively. These images are typical of three such different experiments.

**Figure 4:**
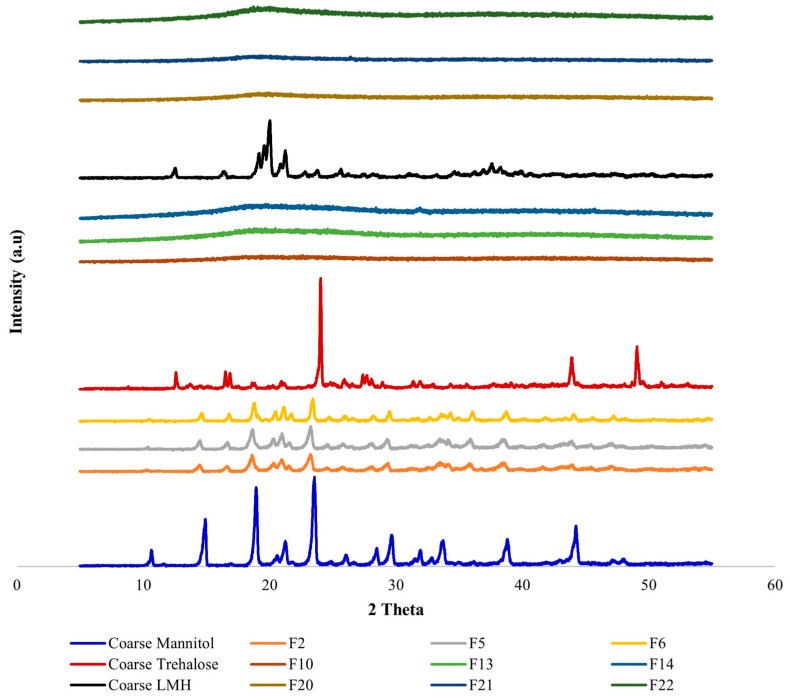
PXRD spectra of coarse carbohydrate carriers, including mannitol, trehalose, and LMH along with their spray-dried powder formulations of SD-mannitol (F2, F5, and F6), SD-trehalose (F10, F13, and F14), and SD-LMH (F20, F21, and F22). These data are typical of three such different experiments.

Upon analysis of SD-mannitol formulations (F2, F5, and F6) using all three airflow rates (357, 473, and 601 L/h), all formulations exhibited smooth, round, and non-agglomerated particles (Figure 3). Moreover, a trend in decreasing particle size was observed (F2 > F5 > F6) with increasing airflow rate (357, 473, and 601 L/h) (Table 3). This may be linked to higher nozzle flow, which may increase the atomization energy exerted on the formulation, hence producing smaller droplets upon spraying, which are further converted into smaller particles upon drying, when compared to lower nozzle flow, which is associated with larger particle sizes (Table 3). SD-mannitol particles were observed to be non-agglomerated, possessing a smooth surface with spherical morphology; this was attributed to the high crystallinity of the carbohydrate carrier (Figure 3). Furthermore, particle sizes for SD-mannitol formulations (F2, F5, and F6) were found to be in the range of 2.96–4.58 µm, demonstrating an ideal size for peripheral deposition within the lungs (Table 3). However, with increasing airflow rates of 473 and 601 L/h (from 357 L/h), both SD-trehalose (F13 and F14) and SD-LMH formulations (F21 and F22) demonstrated particle agglomeration/fusion (Figure 3), when compared to their counterpart formulations with lower airflow rate. This may be related to the amorphous nature of their powder form (Figure 4), where particles possess a propensity to agglomerate due to moisture content in the drying chamber (also observed via decreasing the outlet temperature to 44 and 40 °C for SD-trehalose formulations (F13 and F14, respectively), and to 82 and 79 °C for SD-LMH formulations (F21 and F22, respectively)) (Table 1) (Figure 3). Such formulations where particles are fused/agglomerated are not desirable for peripheral drug deposition. Overall, SD formulations of mannitol (F2, F5, and F6), trehalose (F10), and LMH (F20) were observed to be smooth, spherical with a smaller particle size, and the only non-agglomerated/fused particles.

#### 3.2.2. Effect Airflow Rates on Production Yield, Moisture Content, and Particle Crystallinity

Varying the airflow rates in spray-drying instrument is proposed to affect the drying process as well as the motion of particles during the spray drying process. Upon analysis, it was found that with higher airflow rates of 473 and 601 L/h, respectively, the production yield of SD-mannitol F5 and F6 increased significantly (*p* < 0.05) (i.e., more than 20% from F2 formulations when using an airflow rate of 357 L/h) (Table 3). Moreover, the moisture content of SD formulations also increased (*p* > 0.05) with increasing airflow rate (Table 3). However, due to the crystalline profile of the SD-mannitol formulations (Figure 4), production yield remained high, as particle agglomeration/fusion was not observed.

Similarly, when using a lower airflow rate of 357 L/h, lower production yields of 58.28 ± 4.61 and 70.03 ± 4.29 were observed for SD-trehalose (F10) and SD-LMH (F20) formulations, respectively. However, upon an increase in the airflow rate to 473 and 601 L/h for both SD-trehalose (F13 and F14) and SD-LMH (F21 and F22) formulations, respectively, no significant difference (*p* > 0.05) in the production yield was observed (Table 3). It is suggested that with a lower airflow rate, formulation particles may be retained for longer in the drying chamber, possessing lower moisture content (due to their longer contact time in a drying chamber), whereas with a higher airflow rate, formulation particles may travel faster and stay for a shorter period within the drying chamber and, hence, contain more moisture (Table 3). LMH possesses one water moiety, whereas trehalose possesses two. This difference may increase hydrogen bonding between the carrier and water molecules within the powder, resulting in a higher moisture content for trehalose as opposed to LMH, and this may offer an explanation as to why LMH was more susceptible to changes in airflow. Therefore, employing lower airflow rates (357 L/h) may cause the formulation to reside for longer in the drying chamber and increase the probability of their deposition/sticking on to the walls of the drying chamber, resulting in a lower production yield. Furthermore, higher airflow rates were associated with higher production yield but were also observed to possess a higher moisture content, which yielded agglomerated/fused particles. Analogous results were also noted where a high production yield was obtained when a high airflow rate was employed [29,30]. Both coarse trehalose and coarse LMH exhibited a crystalline profile; however, following spray drying, their crystalline profiles were observed to switch to amorphous (Figure 4); these findings are in accordance with previous studies employing these types of sugars [27,31].

### 3.3. Effect of Pump Feed Rates on the Physicochemical Properties of Spray-Dried (SD) Formulations

The optimum inlet temperature and airflow rate required for spray drying using SD-mannitol (120 °C and 601 L/h), SD-trehalose (120 °C and 357 L/h), and SD-LMH (200 °C and 357 L/h) were identified in Section 2.1 and Section 2.2. Pump feed rate is the volume of formulation injected into the spray-drying instrument for the generation of aerosols, followed by their conversion into dry particles. Three different pump feed rates were employed. These were 5, 15, and 25% (each represented a feed rate of 1.5, 4.5, and 7.5 mL/min, respectively). In this section using spray drying, SD-mannitol (F6, F7, F8), SD-trehalose (F10, F15, F16), and SD-LMH (F20, F23, F24) used a pump feed rate of 15, 5, and 25% (Table 1 and Table 4).

#### 3.3.1. Effect of Pump Feed Rates on Particle Morphology and Particle Size

**Figure 5:**
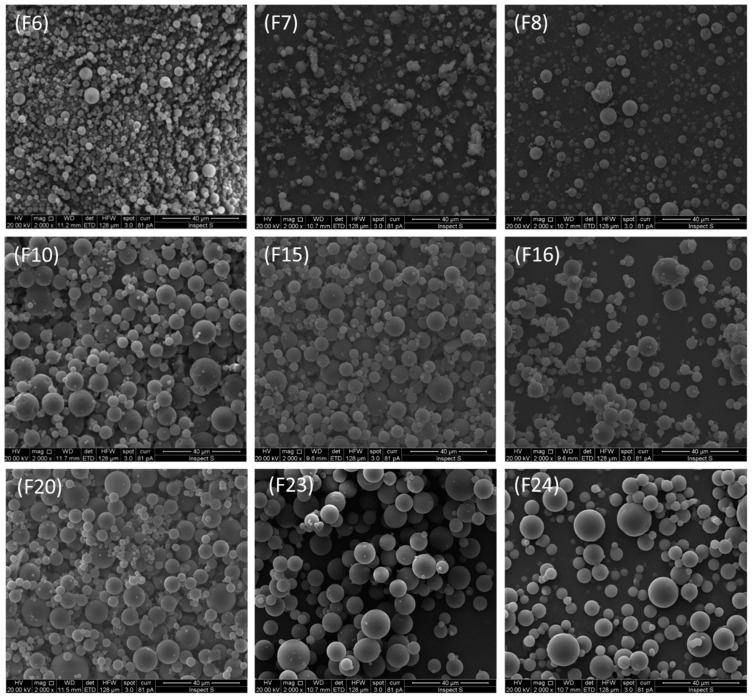
SEM images illustrating the effect of three pump feed rates (15, 5, and 25%) employing a spray-drying instrument on SD-mannitol (F6, F7, and F8), SD-trehalose (F10, F15, and F16), and SD-LMH (F20, F23, and F24) formulations, respectively. These images are typical of three such different experiments.

Based on employing three different pump feed rates (i.e., 15, 5, and 25%), the surface morphologies of SD-mannitol (F6, F7, and F8), SD-trehalose (F10, F15, and F16) and SD-LMH (F20, F23, and F24) formulations were investigated. All the investigated SD formulations exhibited spherical particles with no particle agglomeration or fusion (Figure 5). Therefore, it is suggested that the pump feed rates had no significant (*p* > 0.05) effect on the surface morphology of SD particles. Similarly, the effect of pump feed rates (i.e., 5 to 15 and 25%) demonstrated no significant impact (*p* > 0.05) on particle size, irrespective of SD formulation type. Analogous findings were also found where the feed rate of cellulose suspension in spray drying did not affect the particle size [32]. However, in general, SD formulations produced large particles with a higher pump feed rate (25%) when compared to lower feed rates (5 or 15%) (Table 4).

#### 3.3.2. Effect of Pump Feed Rates on Production Yield, Moisture Content, and Particle Crystallinity

Upon investigation of the effect of pump feed rates (5, 15, and 25%) on the production yield and moisture content, a trend was observed for all three SD-mannitol, SD-trehalose, and SD-LMH formulations (F6, F7, F8; F10, F15, F16; F20, F23, F24). Significantly higher (*p* < 0.05) production yields and lower moisture contents were obtained for a pump feed rate of 5%, whereas a lower yield and higher moisture content were attained using a pump feed rate of 25%, regardless of formulation type (Table 4).

**Figure 6:**
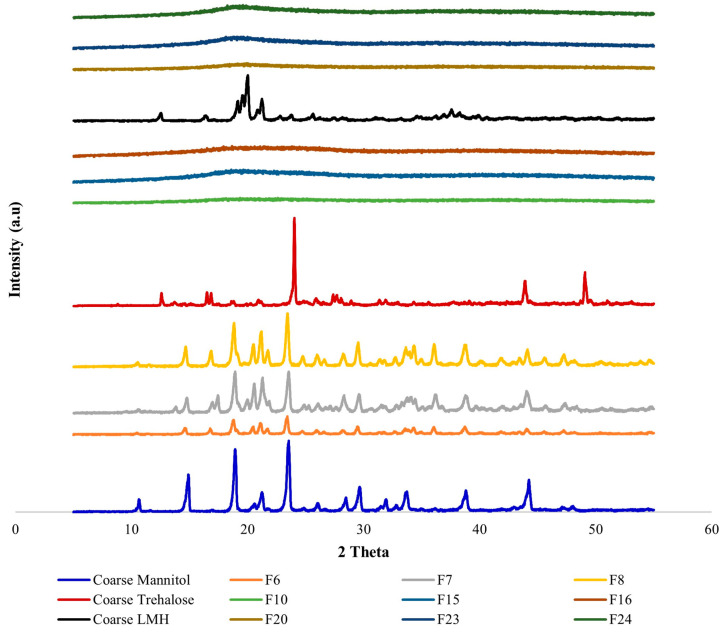
PXRD spectra of coarse carbohydrate carriers, including mannitol, trehalose, and LMH along with their spray-dried powder formulations of SD-mannitol (F6, F7, and F8), SD-trehalose, (F10, F15, and F16), and SD-LMH (F20, F23, and F24). These data are typical of three such different experiments.

The higher production yield at the lower pump feed rate of 5% may be related to the volume of formulation aerosolization and the drying process in the drying chamber of the spray-drying instrument (typically possessing a lower moisture content) (Table 4). The smaller aerosol droplets produced at a lower pump feed rate (5%) in the drying chamber affected the temperature negligibly (i.e., as observed via the outlet temperature; Table 1), where droplets dried fully and much faster into particles (with a reduced number of droplets present in the drying chamber during the spray drying process), whereas, at the higher pump feed rate of 25%, a greater number of aerosol droplets present in the drying chamber correspondingly reduced the chamber temperature (as observed via the lower outlet temperature; Table 1), as well as possessing higher levels of moisture and potentially a higher probability of particle deposition into the internal walls of the drying chamber. Therefore, a trend of lower moisture content and high production yield was observed with decreasing pump feed rate: 5 < 15 < 25% (Table 4). These results are comparable with previous research, where a lower feed rate and lower moisture content of β-galactosidase produced a higher production yield using a spray-drying instrument [33]. Post-spray drying, SD-mannitol (F6, F7, and F8) elicited a crystalline powder with low intensity peaks when compared to the coarse mannitol powder, whereas SD-trehalose (F10, F15, and F16) and SD-LMH (F20, F23, and F24) exhibited amorphous particles (Figure 6). Changes in the PXRD profile for all three sugars before and after spray drying are in agreement with previous literature [27,31]. It is noteworthy that varying the pump feed rate had no observable effect on the characteristic profile of these SD sugars. Thus, based on the higher production yield and lower moisture content, SD-mannitol (F7), SD-trehalose (F15), and SD-LMH (F23) were identified as the most appropriate formulations using a lower pump feed rate of 5%.

### 3.4. Optimized Spray-Dried Proliposome (SDP) Formulation Using DMPC as a Phospholipid

Based on the aforementioned characterization of SD formulations (Section 2.1, Section 2.2 and Section 2.3), optimized parameters (inlet temperature, airflow rate, and pump feed rate) were identified for SD-mannitol, SD-trehalose, and SD-LMH. These optimal parameters were then employed to prepare novel spray-dried proliposome (SDP) formulations at a large scale using mannitol, trehalose, and LMH as carbohydrate carriers (Table 5), where beclomethasone dipropionate (BDP) was employed as a model drug and dimyristolyphosphatidylcholine (DMPC) was used as a phospholipid.

#### 3.4.1. Morphology of Spray-Dried Proliposome (SDP) Formulations

**Figure 7:**
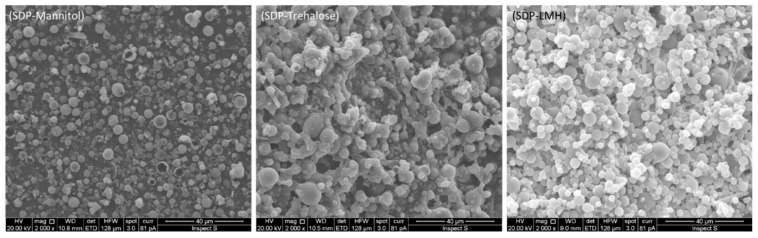
SEM images demonstrating the surface morphology of spray-dried proliposome (SDP) formulations of SDP-mannitol, SDP-trehalose, and SDP-LMH using an optimal inlet temperature (120, 120, and 200 °C, respectively), airflow rate (601, 357, and 357 L/h, respectively) and pump feed rate (15, 5, and 15%, respectively). These images are typical of three such different experiments.

SDP-mannitol particles were observed to be spherical individual particles regardless of phospholipid and drug concentration (Figure 7); this is in agreement with previous findings when using the same carrier [19]. SDP-mannitol formulations were observed to possess a smooth surface with no particle agglomeration/fusion. Similar to SDP-mannitol, the SDP-LMH exhibited a similar pattern in terms of surface morphology, with smooth-surfaced spherical particles, however, showing slight particle agglomeration (Figure 7). Contrasting findings were described by Omer et al. [27], where post-spray drying of LMH proliposome formulations, rough and irregular particles were obtained. Moreover, spherical non-agglomerated particles possess a high chance of deposition in the peripheral airways, particularly when the aerodynamic size ranges between 0.5–5 µm [34,35]. Conversely, SDP-trehalose formulations demonstrated spherical to oval shaped particles with high levels of particle agglomeration (Figure 7). This agglomeration may be attributed to the presence of phospholipid and may justify their low production yield (related to the sticking of particles to the internal walls of drying chamber during spray drying) (Table 5). Furthermore, particle agglomeration may be related to the high surface energy of the SD particles, which is associated with a tendency to increase cohesiveness and, hence, compromise flowability [36]. Agglomerated particles may potentially reduce deep lung deposition; therefore, SDP-mannitol and SDP-LMH are deemed to be more suitable for pulmonary delivery using DPI devices, when compared to SDP-trehalose formulations.

#### 3.4.2. Production Yield, Moisture Content, and Particle Size Analysis of SDP Formulations

As optimized spray drying parameters were employed for all three carbohydrate carriers (mannitol, trehalose, and LMH) to obtain SDP formulations (Table 5), any differences in terms of production yield may be attributed to the carrier type and their interaction with the employed phospholipid. Additionally, the inlet temperature, airflow rate, pump feed rate, and atomizer design may also influence production yield. Production yield was significantly higher (*p* < 0.05) for both SDP-mannitol and SDP-LMH formulations, when compared to the SDP-trehalose formulation (Table 5). The lower production yield of SDP-trehalose may be related to particle agglomeration and their adherence to the inner walls of the spray drying compartments due to the presence of a high moisture content, when compared to SDP-mannitol and SDP-LMH formulations (Table 5). This may be further attributed to the behaviour of the phospholipid (DMPC) with trehalose, where trehalose particles may be unable to uptake the phospholipid adequately within the SD particles, and where the presence of phospholipid on the particles’ surface may lead to bridge formation with the adjacent particles, resulting in particle agglomeration/fusion or large particle formation (Table 5) (Figure 7).

Higher production yield is indirectly related to the particle size and moisture content, where a trend was observed with lower particle size and lower moisture content producing higher yield, and with large particle sizes and high moisture contents producing a lower production yield (Table 5). Thus, both particle size and moisture content significantly affect the production yield. In addition, all three SDP formulations demonstrated higher entrapment efficiency (>94%) for BDP (a lipophilic drug). Overall, SDP-mannitol and SDP-LMH formulations produced a high production yield, small particle size and high entrapment efficiency, making them suitable carriers for pulmonary drug delivery using DPIs.

#### 3.4.3. PXRD of SDP Formulations

**Figure 8:**
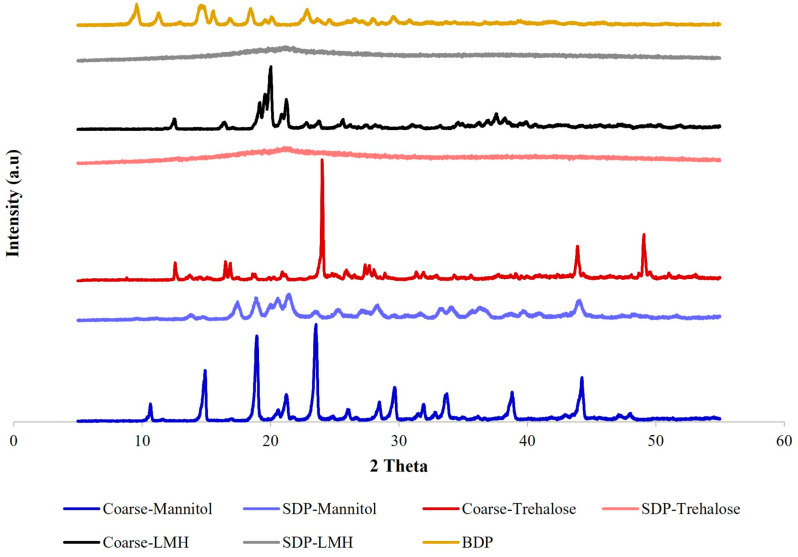
PXRD profile of coarse carbohydrate carriers, including mannitol, trehalose, LMH, and a model drug beclomethasone dipropionate (BDP) along with their spray-dried proliposome (SDP) formulations of SDP-mannitol, SDP-trehalose, and SDP-LMH. These data are typical of three such different experiments.

Prior to spray drying, coarse mannitol, coarse trehalose, and coarse LMH demonstrated high crystallinity; however, post-spray drying, the intensity of the peaks reduced in SDP-mannitol formulations (indicating the reduced crystallinity of this sugar), and no peaks were found for the SDP-trehalose and SDP-LMH formulations (indicating their amorphous form) (Figure 8). The PXRD profile of BDP was not detected in the SDP formulations prepared from mannitol, trehalose, and LMH (Figure 8), which may be related to the lower quantity of BDP employed in formulations as compared to coarse carbohydrate carriers and phospholipids. Moreover, BDP and phospholipids (i.e., DMPC) are lipophilic, and their dissolution in ethanol followed by organic solvent evaporation may keep resulting in retention of the amorphicity of the drug. Similar results were observed when a physical mixture of phospholipid, carbohydrate carrier (i.e., mannitol, sorbitol. or LMH), and BDP at 50 mol% was used to determine the presence of drug peaks via PXRD analysis, whereas the same formulation in proliposome form showed the absence of the BDP peak [1].

#### 3.4.4. Deposition of SDP Formulations Using NGI

Post-aerosolization, the values of ED were significantly higher (*p* < 0.05) for SDP-mannitol and SDP-LMH, when compared to SDP-trehalose, demonstrating superior aerosolization efficiency (Table 6). This may be related to the smaller particle size with spherical morphology of these formulations, resulting in superior delivery from the capsules. Upon analysis of FPD, a trend of increasing BDP deposition in the NGI was observed (SDP-mannitol > SDP-LMH > SDP-trehalose) (Table 6). Both SDP-mannitol and SDP-LMH showed significantly higher BDP deposition in the NGI stages, when compared to SDP-trehalose formulations, demonstrating deep lung deposition, which is considered desirable for such formulations. The agglomerated particles of SDP-trehalose formulations demonstrated high resistance to particle flow in the NGI stages. The presence of phospholipid on the surface (Figure 7) may result in greater adherence of particles in the induction port and pre-separator components of the NGI instrument. Furthermore, this is also confirmed by the lower ED of SDP-trehalose formulations. FPF is the fraction of particles smaller than 5 µm related to the emitted mass of the investigated formulation. A similar trend in terms of FPD was also observed in the SDP-formulations for FPF (Table 6). When employing an airflow rate of 60 L/min, a significantly higher RF was noted for SDP-mannitol formulations (i.e., 86.44 ± 4.55), while this was lower for SDP-trehalose formulations (52.92 ± 5.03). Both SDP-mannitol and SDP-LMH deposited higher levels of RF in the NGI stages, with particle sizes smaller than 5 µm (particle sizes lower than 5 µm are considered a respirable fraction). The trend observed in RF is mirrored in ED and FPD (Table 6).

Mass median aerodynamic diameter (MMAD) is a critical parameter which is known to affect particle deposition in the various stages of NGI and, hence, in the respiratory tract. MMAD is the aerodynamic diameter at which 50% of the aerosolized formulation mass are smaller than the specified average diameter (typically, MMAD should be lower than 5 µm) for their ability to reach and deposit in the peripheral airways. A trend of smaller to higher particle size for MMAD was observed for the SDP-mannitol formulations, followed by SDP-LMH and lastly SDP-trehalose formulations (Table 6). Upon examination of the MMAD values, all three SDP formulations demonstrated a deposition of less than 5 µm. This means that a MMAD less than 5 µm is the acceptable cut-off point for the deposition of formulations in the peripheral region, indicating that all three SDP formulations were highly efficient. Moreover, it is established that the lower the particle size, the better the deposition in the alveolar region of the respiratory tract. Thus, the SDP-mannitol formulation exhibited a MMAD of a smaller size than the counterpart formulations. This was further confirmed by BDP deposition in the alveolar region from the values obtained for FPD, FPF, and RD (Table 6). Similar to MMAD, geometric standard deviation (GSD) demonstrated the same trend of higher to lower values: SDP-mannitol > SDP-LMH > SDP-trehalose. Overall, it was found that SDP-mannitol formulation is an ideal formulation for targeting peripheral lung deposition.

The study findings are promising, suggesting that industrial-scale manufacture of proliposome formulations using the spray drying method is viable, supported by the efficiency of the process using various parameters, as well as the resultant high production yield and desirable formulation properties (amorphous powders with a small particle size and low moisture content suitable for storage).

## 4. Conclusions

In this study, three carbohydrate carriers, namely mannitol, trehalose, and LMH, were used to prepare 24 spray-dried (SD) formulations employing various parameters of the spray-drying instrument, including 4 inlet temperatures (80, 120, 160, and 200 °C), 3 airflow rates (357, 473, and 601 L/h), and 3 pump feed rates (5, 15, and 25%), in order to identify optimum spray drying parameters for these carriers. Only three SD formulations, namely SD-mannitol (F6), SD-trehalose (F15), and SD-LMH (F20) were selected on the basis of their higher production yield, smaller particle size, and lower moisture content, using various inlet temperatures (120, 120, and 200 °C, respectively), airflow rates (601, 357, and 357 L/h, respectively), and pump feed rates (15, 5, and 15%, respectively). Moreover, SD-mannitol formulations showed lower intensity peaks when compared to the crystalline structure of the coarse mannitol using PXRD. Similarly, both coarse trehalose and coarse LMH were found to be crystalline; however, their SD formulations were found to be amorphous, due to the nature of their respective carbohydrate carriers. These optimum parameters of the SD formulations were employed for the preparation of spray-dried proliposome (SDP) formulations, i.e., SDP-mannitol, SDP-trehalose, and SDP-LMH, using DMPC as a phospholipid and BDP as a model drug. Amongst the SDP formulations, SDP-mannitol exhibited a higher production yield, followed by SDP-LMH, whereas a significantly lower yield was noted for the SDP-trehalose formulation. Additionally, the particle sizes and moisture contents of the SDP formulations differed from each other, and were found in the increasing order of SDP-mannitol < SDP-LMH < SDP-trehalose, demonstrating that SDP-mannitol-based formulations are superior. Upon comparison to the SDP-trehalose, and SDP-LMH formulations, the SDP-mannitol was notably efficient in depositing higher FPD, FPF, and RF, and lower MMAD, in the NGI stages when a 60 L/min airflow rate was employed. The SDP-mannitol formulation was suggested to be significantly better in terms of aerosolization performance overall.

## Figures and Tables

**Table 1:** Spray-dried (SD) powder formulations (F1–F24), employing three different carbohydrate carriers, airflow and pump feed rates, and four different inlet temperatures, n = 3.

Formulation	Carbohydrate Carrier	Inlet Temperature (°C)	Outlet Temperature (°C)	Airflow Rate (L/h)	Pump Feed Rate (%)	Aspirator (%)
F1	Mannitol	80	35	357	15	100
F2	Mannitol	120	54	357	15	100
F3	Mannitol	160	73	357	15	100
F4	Mannitol	200	85	357	15	100
F5	Mannitol	120	52	473	15	100
F6	Mannitol	120	51	601	15	100
F7	Mannitol	120	75	357	5	100
F8	Mannitol	120	46	357	25	100
F9	Trehalose	80	40	357	15	100
F10	Trehalose	120	50	357	15	100
F11	Trehalose	160	61	357	15	100
F12	Trehalose	200	65	357	15	100
F13	Trehalose	120	44	473	15	100
F14	Trehalose	120	40	601	15	100
F15	Trehalose	120	65	357	5	100
F16	Trehalose	120	48	357	25	100
F17	LMH	80	35	357	15	100
F18	LMH	120	53	357	15	100
F19	LMH	160	73	357	15	100
F20	LMH	200	84	357	15	100
F21	LMH	200	82	473	15	100
F22	LMH	200	79	601	15	100
F23	LMH	200	87	357	5	100
F24	LMH	200	80	357	25	100

**Table 2:** The effect of four inlet temperatures (80, 120, 160, and 200 °C) using three carbohydrates (i.e., mannitol, trehalose, and LMH) employing spray-dried (SD) mannitol formulations (F1, F2, F3, and F4), spray-dried (SD) trehalose formulations (F9, F10, F11, and F12), and spray-dried (SD) LMH formulations (F17, F18, F19, and F20) on the production yield (%), particle size (µm), and moisture content (%). Data are mean ± standard deviation (STD), n = 3.

Formulations	SD Conditions Inlet Temp (°C), Airflow Rate (L/h), Pump Feed Rate (%)	Production Yield (%)	Particle Size (µm)	Moisture (%)
Mannitol				0.09 ± 0.01
F1	80, 357, 15	38.17 ± 2.61	5.64 ± 3.61	0.33 ± 0.03
F2	120, 357, 15	60.73 ± 4.12	4.58 ± 0.91	0.22 ± 0.03
F3	160, 357, 15	63.64 ± 2.35	5.69 ± 2.72	0.16 ± 0.02
F4	200, 357, 15	55.57 ± 6.64	7.82 ± 2.12	0.14 ± 0.02
Trehalose				9.74 ± 1.43
F9	80, 357, 15	14.63 ± 3.32	Fused particles	7.22 ± 1.13
F10	120, 357, 15	58.28 ± 4.61	4.74 ± 0.95	5.12 ± 0.62
F11	160, 357, 15	58.94 ± 4.26	6.95 ± 1.46	3.54 ± 0.47
F12	200, 357, 15	74.37 ± 4.33	8.27 ± 2.23	1.75 ± 0.85
LMH				5.09 ± 1.25
F17	80, 357, 15	27.26 ± 2.72	Fused particles	4.92 ± 0.84
F18	120, 357, 15	52.68 ± 1.46	Fused particles	4.49 ± 0.92
F19	160, 357, 15	65.34 ± 2.35	6.92 ± 1.52	3.82 ± 0.88
F20	200, 357, 15	70.03 ± 4.29	5.16 ± 1.23	1.99 ± 0.77

**Table 3:** The effect of three airflow rates (357, 473, and 601 L/h) using three carbohydrates (i.e., mannitol, trehalose, and LMH) employing spray drying on the production yield and particle size of spray-dried (SD) formulations. Data are mean ± STD, n = 3.

Formulations	SD Conditions Inlet Temp (°C), Airflow Rate (L/h), Pump Feed Rate (%)	Production Yield (%)	Particle Size (µm)	Moisture (%)
Mannitol				0.09 ± 0.01
F2	120, 357, 15	60.73 ± 4.12	4.58 ± 0.91	0.22 ± 0.03
F5	120, 473, 15	84.57 ± 4.53	4.29 ± 0.51	0.24 ± 0.02
F6	120, 601, 15	86.01 ± 3.25	2.96 ± 0.45	0.25 ± 0.03
Trehalose				9.74 ± 1.43
F10	120, 357, 15	58.28 ± 4.61	4.74 ± 0.95	5.12 ± 0.62
F13	120, 473, 15	64.55 ± 4.34	Fused particles	5.22 ± 0.81
F14	120, 601, 15	60.72 ± 4.96	Fused particles	5.24 ± 1.01
LMH				5.09 ± 1.25
F20	200, 357, 15	70.03 ± 4.29	5.16 ± 1.23	1.99 ± 0.77
F21	200, 473, 15	73.54 ± 7.61	Fused particles	4.05 ± 0.79
F22	200, 601, 15	72.65 ± 6.73	Fused particles	4.13 ± 0.87

**Table 4:** The effect of the three pump feed rates (15, 5, and 25%) using three carbohydrates (i.e., mannitol, trehalose, and LMH) employing spray drying on the production yield and particle size of spray-dried (SD) formulations. Data are mean ± standard deviation (STD), n = 3.

Formulations	SD Conditions Inlet Temp (°C), Airflow Rate (L/h), Pump Feed Rate (%)	Production Yield (%)	Particle Size (µm)	Moisture (%)
Mannitol				0.09 ± 0.01
F6	120, 601, 15	86.01 ± 3.25	2.96 ± 1.42	0.25 ± 0.03
F7	120, 601, 5	88.41 ± 0.64	3.17 ± 1.41	0.20 ± 0.02
F8	120, 601, 25	78.41 ± 3.02	4.07 ± 1.59	0.28 ± 0.03
Trehalose				9.74 ± 1.43
F10	120, 357, 15	58.28 ± 4.61	4.74 ± 0.95	5.12 ± 0.62
F15	120, 357, 5	72.55 ± 5.42	4.55 ± 0.46	3.76 ± 0.75
F16	120, 357, 25	23.71 ± 4.67	4.89 ± 0.32	5.54 ± 0.84
LMH				5.09 ± 1.25
F20	200, 357, 15	70.03 ± 4.29	5.16 ± 1.32	1.99 ± 0.77
F23	200, 357, 5	73.69 ± 2.48	7.48 ± 3.04	1.55 ± 0.31
F24	200, 357, 25	56.17 ± 3.98	7.76 ± 3.18	3.38 ± 0.67

**Table 5:** Spray-dried proliposome (SDP) formulations of SDP-mannitol, SDP-trehalose, and SDP-LMH were prepared using spray drying optimized parameters, including inlet temperature (°C), airflow rate (L/h), and pump for the production yield (%), moisture content (%), particle size (µm), and entrapment efficiency (%). Data are mean ± SD, n = 3.

Formulation	SDP-Mannitol	SDP-Trehalose	SDP-LMH
**Inlet temp (°C)**	120	120	200
**Outlet temp (°C)**	57	74	98
**Airflow rate (L/h)**	601	357	357
**Pump feed rate (%)**	15	5	15
**Production yield (%)**	82.45 ± 4.58	69.54 ± 3.23	77.33 ± 3.75
**Moisture content (%)**	0.22 ± 0.01	2.65 ± 0.06	1.52 ± 0.04
**Particle size via SEM (µm)**	2.64 ± 1.51	4.83 ± 2.26	4.19 ± 2.11
**Entrapment efficiency (%)**	98.51 ± 2.17	94.89 ± 4.26	96.06 ± 4.67

**Table 6:** Aerosolization performance of spray-dried proliposome (SDP) formulations of SDP-mannitol, SDP-trehalose, and SDP-LMH employing emitted dose (ED), fine particle dose (FPD), fine particle fraction (FPF), respirable fraction (RF), mass median aerodynamic diameter (MMAD), and geometric standard deviation (GSD) using a next-generation impactor (NGI). Data are mean ± SD, n = 3.

Formulations	SDP-Mannitol	SDP-Trehalose	SDP-LMH
ED (%)	96.28 ± 3.14	89.63 ± 2.21	97.32 ± 3.35
FPD (µg)	285.81 ± 8.42	153.71 ± 6.53	211.19 ± 7.74
FPF (%)	56.84 ± 4.52	23.76 ± 4.42	44.27 ± 4.77
RF (%)	86.44 ± 4.55	52.92 ± 5.03	77.68 ± 4.19
MMAD (µm)	2.89 ± 0.13	4.98 ± 0.12	4.02 ± 0.14
GSD	2.44 ± 0.03	1.84 ± 0.02	1.96 ± 0.02

## Data Availability

The original contributions presented in this study are included in the article. Further inquiries can be directed to the corresponding author.
